# Waterborne Toxoplasmosis, Brazil, from Field to Gene

**DOI:** 10.3201/eid1202.041115

**Published:** 2006-02

**Authors:** Lenildo de Moura, Lilian Maria Garcia Bahia-Oliveira, Marcelo Y. Wada, Jeffrey L. Jones, Suely H. Tuboi, Eduardo H. Carmo, Walter Massa Ramalho, Natal J. Camargo, Ronaldo Trevisan, Regina M.T. Graça, Alexandre J. da Silva, Iaci Moura, J.P. Dubey, Denise O. Garrett

**Affiliations:** * Ministério de Saúde, Brasília, Brasil;; † Universidade Estadual do Norte Fluminense Darcy Ribeiro, Rio de Janeiro, Brazil;; ‡ Centers for Disease Control and Prevention, Atlanta, Georgia, USA;; § Secretaria de Saúde do Estado do Paraná, Curitiba, Brazil;; ¶ Laboratório Central de Saúde Pública, Curitiba, Brazil;; # United States Department of Agriculture, Beltsville, Maryland;; ** Centers for Disease Control and Prevention Foundation, Atlanta, Georgia, USA

**Keywords:** *Toxoplasma gondii*, outbreak, water, dispatch

## Abstract

Water was the suspected vehicle of *Toxoplasma gondii* dissemination in a toxoplasmosis outbreak in Brazil. A case-control study and geographic mapping of cases were performed. *T. gondii* was isolated directly from the implicated water and genotyped as SAG 2 type I.

Water has been considered an important vehicle for disseminating human toxoplasmosis in outbreaks (*1**,**2*) and in endemic settings in Brazil (*3*). We investigated a large toxoplasmosis outbreak in which the exposure to known sources of *Toxoplasma gondii* infection was assessed. We found that unfiltered, municipally treated water was the epidemiologically implicated source of infection for this outbreak. Isolation, polymerase chain reaction (PCR) detection, and genotyping of *T. gondii* from the implicated water source were demonstrated.

## The Study

In November 2001, in Santa Isabel do Ivai, (southern state of Paraná), a local physician requested serologic tests to diagnose dengue, mononucleosis, cytomegalovirus infection, hepatitis, and toxoplasmosis in 2 persons in whom fever, headache, and myalgias had developed. Positive results were obtained for anti–*T. gondii* immunoglobulin M (IgM) and IgG only. Through the end of 2001, 294 similar cases, which were serologically confirmed as toxoplasmosis, were reported to health authorities in the same area.

**Figure 1:**
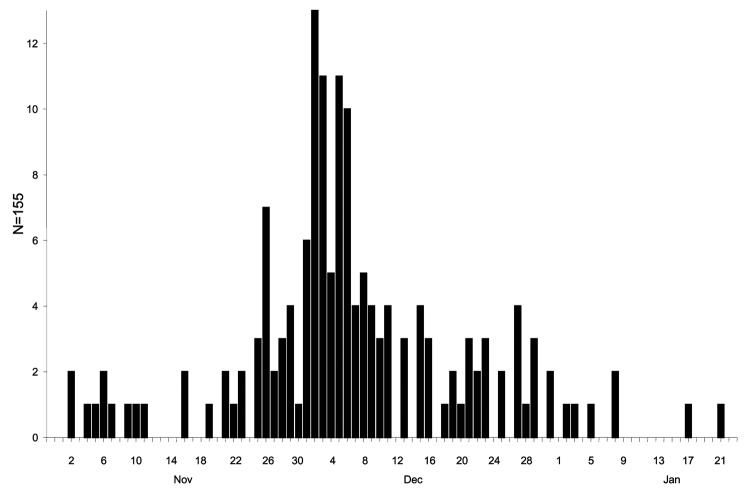
Epidemic plot of the 155 cases registered from November 2001 to January 2002. The dates of the initial symptoms are known only for the 155 individuals among 156 who participated in the case control study.

The outbreak peaked between November 2001 and January 2002 (Figure 1). Symptoms were reported by 155 persons; the main symptoms were headache (n = 135), fever (n = 128), malaise (n = 128), myalgia (n = 124), lymphadenitis (n = 117), anorexia (n = 107), arthralgia (n = 95), night sweats (n = 83), vomiting (n = 60), and rash (n = 11). The duration and magnitude of the epidemic curve could have been influenced by the intensity of media reporting at specific times, which led to people seeking toxoplasmosis testing.

Case-patients were located by active contacting of and passive reporting from local physicians, and media campaigns (television, radio, and newspapers). A matched case-control study was conducted from January 15 to February 2, 2002. Acute cases were defined by standard serologic criteria (*4*) and were selected from a list of volunteers. A total of 2,884 of 6,771 persons living in the urban area of the city volunteered to be serologically tested. A total of 426 (11.5%) persons had anti–*T. gondii* IgM and IgG antibodies; 1,255 (51%) were positive only for IgG antibodies. Of 426 persons who had anti–*T. gondii* IgM and IgG antibodies, 176 met the case definition; of these, 156 (89%) participated in the case-control study. Sex and age matched controls (±5 years, n = 220) were selected from the same group of volunteer who were asymptomatic and seronegative for *T. gondii*.

Serum samples from case-patients and controls were tested for anti–*T. gondii* IgM and IgG antibodies by the Central Laboratory of the Paraná State by using 3 different commercially available enzyme-linked immunosorbent assays (ELISAs) because it was not possible for a single vendor to provide the number of required kits. Fifty percent of the case serum samples (78 samples of 156 participants) were randomly retested in a toxoplasmosis serology reference laboratory, Laboratory of Protozoology at the Tropical Medicine Institute of São Paulo. Five (6.4%) IgM- and IgG-positive serum samples, tested previously with 1 of the commercial kits, showed very low IgG avidity when tested by this laboratory. All the other serum sample test results were confirmed by testing conducted in this laboratory.

**Figure 2:**
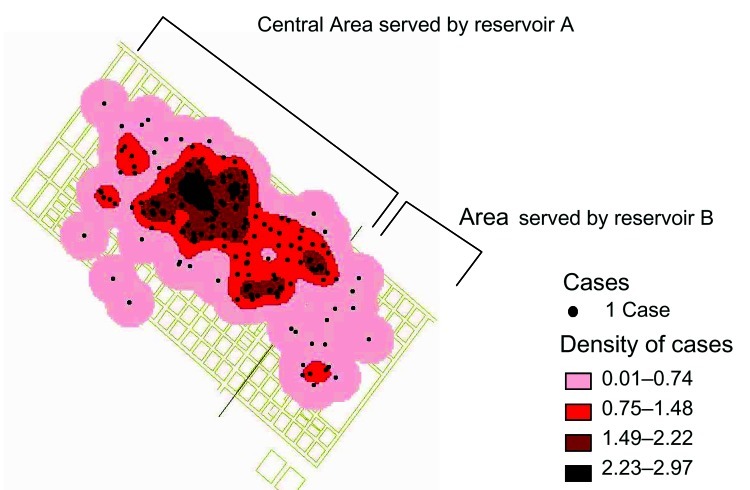
Spatial distribution in km^2^ of the 176 cases that met the case definition. The number of cases is higher in the central area than in the periphery. The reservoir tanks served 2 different parts of the city as depicted by the letters A and B. Water samples from reservoir B, which was considered not implicated in the outbreak, were not investigated; during the water sample collection period (January 9–18), there were no identified household tanks served by reservoir B that had stored water that had been distributed during the outbreak peak.

Of the 156 participants, 138 (88%) lived in the area served by reservoir A and 17 individuals lived in area served by reservoir B (Figure 2); 1 person had a private well. Table 1 shows the univariate analysis results and Table 2 shows the multivariate analysis results. Case-patients were significantly more likely than controls to drink water supplied by municipal reservoir A than reservoir B, as well as to eat commercial ice cream than not. The 4 case-patients that reported not drinking water from reservoir A, however, reported eating ice cream. The frequency of eating ice cream among the persons who drank water from the reservoir A was 32%.

**Table 1:** Univariate analysis showing risk factors for *Toxoplasma gondii* infection for statistically significant factors (positive results only), N = 376

Characteristic	No. persons*	Case	Control	Matched odds ratio	p value	95% confidence interval
Water exposure						
	Drank water exclusively from municipal tank reservoir				3.73	0.016	1.27–10.93
	A	350	152	198			
	B	26	4	22			
	Household tank				2.16	0.006	1.24–4.01
	No	95	28	67			
	Yes	281	128	153			
	Drank >10 cups water per day				2.07	0.004	1.24–3.61
	No	270	97	173			
	Yes	106	59	47			
	Drank beverages made with unfiltered water				2.25	0.044	1.02–5.50
	No	34	20	14			
	Yes	342	136	206			
Food exposure						
	Ate undercooked meat in past 30 days				2.71	0.027	1.11–7.34
	No	345	136	209			
	Yes	31	20	11			
	Ate commercial ice cream				3.43	0.000	2.08–5.67
	No	188	51	137			
	Yes	188	105	83			
	Ate bacon				1.89	0.009	1.15–3.02
	No	228	82	146			
	Yes	148	74	74			
	Ate lamb				1.85	0.043	1.02–3.51
	No	316	122	194			
	Yes	60	34	26			
	Ate in restaurants in the past 30 days				1.71	0.028	1.06–2.96
	No	277	105	172			
	Yes	99	51	48			

*Case-patients ranged from 1 to 72 years of age (median = 28); 79 (51%) were male; 6 (3.8%) were pregnant woman.

**Table 2:** Risk for *Toxoplasma gondii* infection shown as odds ratios estimated with conditional backward elimination logistic regression, N = 376

Variable	Odds ratio	Wald confidence limits		
		Lower	Upper	p value*
Drinking water from reservoir A	4.55	2.01	5.49	0.001
Drinking >10 glasses of water per day	3.29	1.46	4.46	0.001
Having household water storage tank	1.81	0.99	3.33	0.054
Eating commercial ice cream	4.55	2.01	5.49	0.001

*Significant (p<0.001, rounded).

The environmental investigation included mapping the city water supply system which is served by 2 municipal tank reservoirs (reservoir A and reservoir B) that both receive water from underground, protected deep wells. Both reservoirs are tanks with 150,000 L storage capacity. Case distribution showed a clustering in the central area served by reservoir A (Figure 2).

Because the environmental investigations and the case-control study started in parallel on January 9, 2002, and the outbreak had peaked (Figure 1), the chances of detecting parasites in the municipally distributed water were theoretically low. To increase the chances of detecting the parasite in water, household tanks that had water that had been distributed during the outbreak peak were identified. These tanks could be investigated in municipal schools that stopped water use due to summer vacations from December 17, 2001, to the end of January 2002. Despite the risk from eating ice cream (Table 2), no ice cream made during the outbreak period was available for laboratory testing. The ice cream was prepared locally in small batches with water from reservoir A.

We identified 4 schools that had water in their household tanks that had been distributed by reservoir A during the peak of the outbreak. Approximately 4,650 L of water collected from these tanks was filtered through 56 fluoropore membrane filters (Millipore Billerica, MA, USA). We retrieved 19 liters of water concentrated to 60 mL by centrifugation (600 × *g* 30 min 4°C). The membrane filters were divided into 3 equal sets. One set remained in Brazil (Universidade Estadual do Norte Fluminense Darcy Ribeiro) for bioassays in *T. gondii*–seronegative chickens and further genotyping. One set was sent to the US Department of Agriculture for bioassays in *T. gondii*–seronegative pigs and cats, and 1 was sent to the Centers for Disease Control and Prevention for PCR analysis. Chickens and pigs were fed with membrane filters and their serum samples tested by ELISA and or modified agglutination test (*5*) until seroconversion. The seropositive animal organs were examined for *T. gondii* (*6*). Control animals were fed with noncontaminated membrane filters. Water samples from the 4 schools' household tanks were positive for *T. gondii* by at least 1 assay method. Parasites were found in the lungs of mice injected with brain and heart tissue of seropositive chickens. Cats fed pig tissues shed *T. gondii* oocysts after 4–5 days. Oocysts from cat feces were injected into mice, which died of acute toxoplasmosis. Viable *T. gondii* was recovered in mice after subpassage as verified by optical microscopy. The nested amplification of SAG 2 followed by restriction fragment length polymorphism identified type I *T. gondii* from chickens and pigs (*7*).

DNA extraction from fluoropore membranes was performed with the FastDNA extraction method (Qbiogene, Irvine, CA, USA), by using a procedure previously published (*8*), and PCR was performed on extracted DNA by using primers Toxo B22 and B23 (*9*). PCR from DNA extracted directly from the fluoropore membranes was tested blindly by 2 persons on 3 aliquots extracted individually from each membrane filter. The correct size fragment of 115 bp from B1 *T. gondii* gene was amplified from each DNA aliquot extracted from membranes used to process water from 3 of the implicated tanks.

## Conclusions

Our investigation determined that this toxoplasmosis outbreak was associated with consumption of contaminated water, or ice cream prepared with contaminated water, during the outbreak peak. The main factor leading to contamination of reservoir A was the vulnerability to infiltration due to its precarious state of conservation. We propose that reservoir A was contaminated with *T. gondii* oocysts because 1) a female cat living in the reservoir A area delivered 3 kittens in early October 2001; 2) the kittens lived on the top of the tank reservoir; and 3) the kittens were most likely weaned by the first week of November. However, it was not possible to confirm *T. gondii* in the kittens because we were not able to catch them. The reservoir shelter roof tiles were removed and not replaced until the end of heavy summer rains. From November 4 to December 12, the daily rainfall varied from 27 mm to 72 mm. Reservoir A, constructed in the 1940s, had cracks that were unprotected from rain water, which were likely contaminated with cat feces. These factors could have been enhanced by the lack of filtration and flocculation processes as part of the water treatment. Additionally, the level of chlorination used to treat water in municipal systems is inadequate to eliminate *T gondii* oocysts (*10*).

Of the 408 case-patients examined for ophthalmologic conditions through February of 2002 who were *Toxoplasma* IgM and IgG positive, 10% had ocular lesions; however, only 4.4% had necrotizing retinal lesions (*11*). The frequency of symptoms observed in this study may be associated with the dose and virulence of organisms ingested since parasites of genotype I, which are of high virulence (*12**,**13*), were isolated from the water implicated in the outbreak. These data are consistent with other studies also showing SAG-2 type I parasites isolated from the environment from different geographic areas in Brazil (*14*), including in the outbreak area (*15*). Demonstration of the parasite in the outbreak implicated water was decisive in the closing of reservoir A and the construction of a new municipal reservoir.
